# Diagnosing Burkitt Lymphoma in Sub-Saharan Africa by Sequencing of Circulating Tumor DNA: A Comparative Microcosting Study

**DOI:** 10.1016/j.vhri.2025.101113

**Published:** 2025-07

**Authors:** Liz Morrell, Malale Tungu, Caroline Achola, Ismail Legason, Erick Magorosa, Priscus Mapendo, Leah Mnango, Alex Mremi, Heavenlight Christopher, Emmanuel Josephat, Adam Burns, Helene Dreau, Mihaela Leonte, Lulu Chirande, Salama Mahawi, Elifuraha Mkwizu, Hadija Mwamtemi, Godlove Sandi, Claire El Mouden, Anna Schuh, George Ruhago, Sarah Wordsworth, AI-REAL Consortium, Caroline Achola, Pamela Atim, Adam Burns, Clara Chamba, Lulu Chirande, Faraja Chiwanga, Anthony Cutts, Helene Dreau, Claire El Moulden, Edrick M. Elias, Philomena Goodluck, Oliver Henke, Kieran Howard, Jingjing Jiang, Daisy Jennings, Emmanuel Josephat, Atukuzwe Kahakwa, Hadija Kaliisa, Jacqueline Kamanga, Ismail D. Legason, Mihaela Leonte, Laura Lopez Pascua, Erick Marogosa, Salama Mahawi, Priscus Mapendo, William F. Mawalla, Sam M. Mbulaiteye, Daniel Mbwambo, Elifuraha Mkwizu, Leah Mnango, Liz Morrell, Alex Mremi, Hadija Mwamtemi, Liberata Mwita, Martin D. Ogwang, Isaac Otim, Kate Ridout, George Ruhago, Godlove Sandi, Raphael Sangeda, Patricia Scanlan, Kristin Schroeder, Anna Schuh, Rehema Shungu, Paul Shadrack Ntemi, Malale Tungu, Dimitris Vavoulis, Sarah Wordsworth

**Affiliations:** 1 Health Economics Research Centre, Nuffield Department of Population Health, University of Oxford, Oxford, England, UK; 2 School of Public Health, Muhimbili University of Health and Allied Sciences, Dar es Salaam, Tanzania; 3 Central Public Health Laboratory, Kampala, Uganda; 4 Department of Oncology, University of Oxford, Oxford, England, UK; 5 Department of Pathology, Muhimbili National Hospital, Dar es Salaam, Tanzania; 6 Department of Pathology, Kilimanjaro Christian Medical Centre, Moshi, Tanzania; 7 Department of Haematology and Blood Transfusion, Muhimbili University of Health and Allied Sciences, Dar es Salaam, Tanzania; 8 Department of Pediatrics, Muhimbili National Hospital, Dar es Salaam, Tanzania; 9 Department of Oncology, Kilimanjaro Christian Medical Centre, Moshi, Tanzania; 10 National Institute for Health Research Oxford Biomedical Research Centre, University of Oxford, Oxford, England, UK

**Keywords:** Burkitt lymphoma, circulating tumor DNA, diagnosis, microcosting, sub-Saharan Africa

## Abstract

**Objectives:**

Determining the cost of diagnosis of Burkitt lymphoma by DNA sequencing from a blood sample, compared with current histopathology. Estimating future sequencing costs at increased scale and exploring the effect of positivity rate on per-case cost.

**Methods:**

We conducted a microcosting of both diagnostics. Resource use information was derived from standard operating procedures and interviews with staff. Unit cost data were from salary scales, purchase records, and publicly available prices. Costs were collected during 2021 and 2022, in the currency of purchase, and converted to common year (2024) and currency (US dollar [$]), with a discount rate of 5%. For increased scale, we assumed simple scaling up of current sample preparation and higher-capacity sequencing machines running at least once a week to maintain turnaround times.

**Results:**

We estimated a cost of $185.01 per patient for histopathology, with the main cost drivers being staining ($87.20, largely immunohistochemistry consumables, including $34.52 for antibodies) and the biopsy procedure ($72.29). The cost of the sequencing-based diagnostic was $710.15 at current throughput, with the largest contribution from the sequencing step because of the cost of sequencing reagents ($175.48 per sample). Costs are sensitive to throughput, reagent costs, and efficiency of utilization of equipment. At the current prevalence, cost per positive case is 2-fold higher at a positivity rate of 25% compared with 75%.

**Conclusions:**

With the current technology and throughput, sequencing is likely to increase the cost of diagnosis compared with current pathology. Costs will reduce with increased scale, which requires establishing local reagent supply and maintenance capability.

## Highlights

• Delayed or imprecise diagnosis contributes to the high burden of childhood lymphomas in sub-Saharan Africa. Diagnosis via sequencing of circulating tumor DNA (liquid biopsy) potentially offers rapid and specific diagnosis and avoids the need for invasive tissue biopsy.
• Microcosting found that liquid biopsy costs $525 more per patient than histopathology. These costs reduced with increased scale and strengthened local supply infrastructure.
• Liquid biopsy may nonetheless be cost effective in childhood cancers because many life years could be saved.

## Introduction

Burkitt lymphoma (BL) is a highly aggressive non-Hodgkin lymphoma.^1^^,^^2^ BL is classified by the World Health Organization into endemic, sporadic, and immunodeficiency-related clinical groups^1^^,^^3^; the endemic type is the most common pediatric malignancy in sub-Saharan Africa (SSA) and is linked to infection with the Epstein Barr virus and malaria.^1^^,^^3^

About 90% of children with mature B-cell lymphomas in high-income countries are cured with frontline chemotherapy.^4^^,^^5^ This is achieved through timely and precise diagnosis, risk stratification, highly penetrative chemotherapy, and good supportive care.^6^ In sharp contrast, in SSA, childhood lymphomas represent a high disease burden with estimated cure rates of only 30% to 50%.^6^^,^^7^ These outcomes may be related to delayed or imprecise diagnosis, due to shortages of both surgeons and pathologists and technical challenges of performing high-quality hematopathology.^4^^,^^8^^,^^9^

BL is driven by chromosomal translocations that cause the overexpression of oncogene c-myc.^1^ Sequencing of circulating tumor DNA from a blood sample (known as liquid biopsy) has the potential to deliver a precise diagnosis by identifying such hallmark genetic changes. It may also be faster than traditional pathology and avoids the need for invasive tissue biopsy.^10^ The Aggressive Infection-Related East African Lymphoma study (AI-REAL) aims to validate the use of liquid biopsy in the diagnosis of infection-related lymphomas, including BL.^11^ The study, based in Tanzania and Uganda, is a prospective cohort study among children with suspected lymphoma, and established the first in-country diagnostic sequencing capability for circulating tumor DNA in these countries.

Alongside the clinical validation of liquid biopsy in this context, economic evidence is needed to inform adoption and funding decisions. An economic evaluation will provide evidence of the likely cost-effectiveness of liquid biopsy-based diagnostics in the SSA setting, using BL as an example. As part of the economic evaluation, we undertook a costing of the new diagnostic method in Tanzania, compared with current practice (diagnosis based on histopathology), from a third-party payer’s perspective. Here, we describe the costing, univariate sensitivity analysis, estimates of future costs with increased scale, and consideration of diagnostic cost per positive case detected.

## Methods

### Study Setting

The protocol for the AI-REAL study is published.^11^ Conducted between May 2019 and June 2023 across 4 hospital sites in Tanzania and Uganda, the study aimed to validate the diagnosis of childhood lymphomas using liquid biopsy, compared with current histopathology. Ethical approval was obtained from relevant bodies.^11^ We estimate costs for Tanzania, where the sequencing capability of the study was developed (Muhimbili University of Health and Allied Sciences [MUHAS], Dar-es-Salaam). Histopathology was costed at nearby Muhimbili National Hospital (MNH) for comparability, and because it was the largest hospital in the study, it was the best one resourced to support the work.

### Costing Approach

Costs were derived using microcosting: a detailed method that identifies and quantifies all resources used and the cost of each resource (known as the unit cost: for example, an hour of a doctor’s time, a piece of equipment, or a disposable pipette).^12^^,^^13^ The resource use is multiplied by the associated unit cost. Microcosting has been applied previously to genomic diagnostics in cancer.^14^, ^15^, ^16^ It is an appropriate method to apply in this context, in which the diagnostic method is new in this setting and no published costs or tariffs are available. Our work is informed by costing methods of Drummond et al,^12^ and our reporting follows the costing elements of the Consolidated Health Economic Evaluation Reporting Standards^17^ (see Appendix 1 in Supplemental Materials found at https://doi.org/10.1016/j.vhri.2025.101113) and, specifically for microcosting, the recommendations of Xu et al.^13^

Microcosting was conducted during 2021 (histopathology) and 2022 (liquid biopsy); a schematic overview of data collection and analysis for each diagnostic is shown in Appendix 2 in Supplemental Materials found at https://doi.org/10.1016/j.vhri.2025.101113. Our analysis included resources used for all of the steps of the diagnostic process from taking the sample from the patient (tissue or blood) to producing the diagnostic report. We assumed that other diagnostic procedures (for example, scans for staging purposes) and consultations were independent of the diagnostic approach; therefore, they were excluded from this comparative analysis. The perspective was that of a healthcare third-party payer because the work aimed to quantify the cost to payers of adopting the liquid biopsy diagnostic. Costs were estimated at the current working capacity of the 2 diagnostic approaches.

To identify and the quantify resources used, Laboratory Standard Operating Procedures for each diagnostic approach were used as the basis for costing questionnaires to gather resource use information, specifically, to enumerate all equipment and consumables and the amount of staff time required for each activity. Information was provided by AI-REAL laboratory staff, bioinformaticians, pathologists, and clinicians.

Unit costs of equipment and consumables were taken from laboratory records, supplier invoices and price lists. Equipment costs (for example, a microtome or sequencing machine) were divided across the item’s expected lifetime and depreciated using equivalent annual costing at a discount rate of 5% as recommended for low- and middle-income countries^18^; annual equipment maintenance costs were included. Hourly staff costs were derived from the midpoint of the appropriate salary grade from the relevant institution (see Appendix 3 in Supplemental Materials found at https://doi.org/10.1016/j.vhri.2025.101113) and adjusted for employer taxes and superannuation (13%).

For histopathology, unit costs were collected in local currency; for liquid biopsy, in the currency in which they are quoted (local currency, US dollars [$] or GB pounds[£]), converted to local currency at representative June 2021 exchange rates (£ = 3200 Tanzanian shillings [TZS], $ = 2300 TZS.^19^ Costs were adjusted to 2024 prices using the gross domestic product (GDP) deflator.^20^ Sensitivity analysis is reported as difference from base cost in local currency.

An overhead rate of 20% was applied, to cover costs such as space and shared services, consistent with other microcosting studies involving DNA sequencing.^14^, ^15^, ^16^^,^^21^

Some equipment is donated by suppliers or charitable organizations. Furthermore, some consumables are funded by charitable organizations. These items were included at their market price.

#### Histopathology

Procedure: tumor tissue biopsy samples are prepared as tissue blocks in formalin, which are then microtome-sectioned for staining and microscopic examination. Hematoxylin and eosin stain is used initially to observe the morphology of the tumor cells; further stains with antibodies (immunohistochemistry [IHC]) are then used to reveal presence of diagnostically relevant proteins expressed by the tumor cells.

The tissue biopsy cost was taken from the hospital’s estimate of the cost to public patients. We costed each of the 2 types of tissue biopsy used: a lymph node biopsy and a Trucut biopsy using a specific type of needle (cost of a small biopsy plus the needle). The proportion of the 2 biopsy types was taken from AI-REAL study data.

AI-REAL followed current best-practice diagnosis^22^^,^^23^ including reviewing findings and concurrence on diagnosis or further testing, by 3 pathologists. Because the number of pathologists at each site is limited, microscope slides were scanned and uploaded to a shared site for review. The costing includes a flat-bed scanner for each site plus the time needed for a laboratory assistant to scan and upload and 2 additional pathologists to review.

Costs per stained slide (including the preparation of control samples) were estimated for both hematoxylin and eosin staining and immunohistochemistry and added to provide a cost per patient. The average number of antibody stains per patient, and an average cost of the staining antibodies used, were derived from AI-REAL patient data.

Sample number was assumed to be similar to that in the current diagnostic service (40 samples per day), and testing error rates were estimated by the pathologists. We assumed that the tissue blocks are consumed during the diagnostic process; therefore, no long-term storage is included. Staff training was estimated by study pathologists as 1 month per year for both laboratory scientists and pathologists.

The costing for MNH was reviewed by both a second Tanzanian study site, Kilimanjaro Christian Medical Centre, and the Ugandan site, St Mary’s Hospital Lacor. Both concurred that the resource use estimates appeared reasonable.

#### Liquid biopsy

Procedure: blood is collected from the patient, and cell-free DNA (cfDNA) is isolated from the plasma. The cfDNA is amplified (copied multiple times to enhance the signal) using polymerase chain reaction. Six samples are then combined into a single pool, with each DNA sample tagged for identification. The pooled sample undergoes hybridization capture, which isolates the DNA segments of interest for sequencing. The pooled sample is loaded onto a sequencing machine (Illumina MiSeq), and the data are uploaded to Amazon Web Services for statistical analysis to identify the presence and level of diagnostic sequences in each patient’s sample.

Standard laboratory reagents (for example, ethanol) and consumables (such as disposable pipette tips) were purchased locally. However, for specialist sequencing reagents, the local supply chain was underdeveloped in Tanzania; therefore, these were purchased by Oxford University and shipped to MUHAS. Our analysis used the price quoted to Oxford. Shipping cost was taken from an example shipment to MUHAS, containing sufficient reagents to sequence approximately 60 samples. Resource use in organizing and managing the shipment was estimated by the Oxford and MUHAS staff involved.

We assumed a throughput of 300 samples per year (1 pool of 6 samples per week), consistent with the laboratory’s current throughput and with estimates of BL incidence in East Africa.^24^^,^^25^ Error rates for a typical laboratory providing a diagnostic service were estimated by lead scientists at the Oxford Molecular Diagnostics Centre, a collaborating laboratory at Oxford University. Staff training resource was assumed to be comparable to histopathology for ongoing staff development.

#### Sensitivity analysis

Parameters evaluated in univariate sensitivity analysis are summarized in Table 1. Salary bands for staff grades are provided in Appendix 3 in Supplemental Materials found at https://doi.org/10.1016/j.vhri.2025.101113. Ranges for sensitivity analyses were informed by reported variability in resource use and observed variation in unit costs, such as invoiced prices.

**Table 1:** Parameters varied in univariate sensitivity analysis.

Parameter	Base value	Range for sensitivity analysis	Source
Salaries	Midpoint of scale	Highest and lowest salary in band range	Government and university pay scales
NI and superannuation	13%	10%, 20%	AI-REAL
Discount rate	5%	3.5%, 10%	^18^
Overheads	20%	15%, 25%	Similar microcostings^14^, ^15^, ^16^^,^^21^
Exchange rate: TZS to USD	TZS 2300/$	2225 to 2500 (range seen in past 5 years)	^19^
Exchange rate: TZS to GBP	TZS 3200/£	2500 to 3299 (range seen in past 5 years)	^19^
Equipment life	Lab estimate per item	−5, +5 years (minimum 2 years)	Resource use questionnaires
Equipment cost	Purchase price	−10%, +25%	Purchase records
**Histopathology**			
Number of patients	40 samples per day	−20%, +20%	Resource use questionnaires
Number of immuno-stains per patient	2.4 per patient	−1, +1	AI-REAL
Lab efficiencies: number of slides per run of the automated stainer	20	10 (20 is full capacity)	Resource use questionnaires
Cost of IHC reagents	$23.67 per slide (AI-REAL)	−50%, +50%∗	Purchase records
Cost of open biopsy	TZS 144K	Lymph node biopsy: TZS 115K (NHIF 2016 tariff), TZS 240K (private patient tariff)	NHIF
Cost of Trucut biopsy	TZS 200K	Small biopsy + Trucut needle: TZS 50K, TZS 287K†	NHIF
Time taken for lab work	Lab estimate	−10%, +10%	Resource use questionnaires
Time taken for review	Pathologist/clinician estimate	−10%, +10%	Resource use questionnaires
Requirement for remote review by 2 additional pathologists	Yes	Not required	AI-REAL pathology protocol
**Liquid biopsy**			
Throughput	300 samples per year	100 (recruitment rate in AI-REAL) 900 (maximum in current laboratory)	Resource use questionnaires
Proportion of samples from remote sites	40%	20%, 70%	AI-REAL
Consumables	Purchase records	−20%, +20%	Purchase records
Sourcing strategy	Specialist DNA reagents shipped from Oxford	Hybrid: reagents with a local supply route by end 2023 sourced locally, others shipped from Oxford Local: local sourcing premium 50% relative to trial prices, and no shipping cost	Purchase records, AI-REAL
Reagents shipment size	Reagents for 60 samples	30, 90 samples‡	AI-REAL shipping records
Reagents shipment cost	$3150	±40% shipping cost, salaries at high/low range of grade	AI-REAL shipping records
Data storage duration	5 years	3, 10 years	Resource use questionnaires
Data storage cost	$0.024/GB/month	−20%, +50%§	Resource use questionnaires
Lab efficiencies: sample pooling	6 samples per pool	5 samples per pool (6 is the maximum)	Resource use questionnaires
Lab efficiencies: bioanalyzer chips used	Optimum	+2 chips per pool of samples	Resource use questionnaires
Error rates	Error rates in routine service	Lab estimates: +20%, aspirational standards achievable in high performing lab	Resource use questionnaires
Staff time estimates	Staff estimates	−20%, +20%∥	Resource use questionnaires

*Note.* Costs are shown in the currency in which they were collected.

AI-REAL indicates Aggressive Infection-Related East African Lymphoma study; GBP, GB pound sterling; IHC, immunohistochemistry; NHIF, National Health Insurance Fund, 2016 tariff inflated to 2022 prices; TZS, Tanzanian Shillings; USD, US dollars.

∗ Variation in IHC reagent costs is based on variation seen in invoices and could reflect market conditions or fluctuations in currency because these reagents are imported in dollars.

† Lower bound is TZS 50K NHIF tariff, plus lowest estimate TZS 50K for needle; higher bound is TZS 137K private patient tariff, plus highest estimate TZS 150K for needle.

‡ The cost of the shipping is held constant because the majority of the cost is driven by the need for dry ice and other fixed costs, with little impact of the weight of the reagents.

§ Each patient assumed to require 6 GB (raw data plus analysis files).

∥ Range is wider than used for histopathology because this is a new process not yet established as a routine service.

### Scale-Up Scenarios

The sequencing operation used in this study is small scale in a research laboratory, which will need to expand as sequencing technology becomes more widely adopted in the region. To estimate the effect of expansion on cost, we substituted the costs of using larger sequencing machines (Illumina’s NextSeq and NovaSeq), specifically, the cost of the sequencing machine itself, reagents, and annual maintenance. All other cost inputs remained the same (that is, we assumed simple scaling up of the same sample preparation process, ignoring other potential economies of scale). Estimates of the optimum number of samples per run of each sequencing machine were provided by lead scientists at Oxford Molecular Diagnostics Centre based on their experience with these machines, assuming panel sequencing similar to that used in the AI-REAL study, at a similar specification (see Appendix 4 in Supplemental Materials found at https://doi.org/10.1016/j.vhri.2025.101113). We assumed at least 1 sequencing run per week, 50 weeks per year, to maintain turnaround times. If multiple machines were required to handle the sample volume, all machines were assumed to run full.

### Cost Per Case Detected

Recent estimates indicate 171 cases of BL in Tanzania during 2018^25^ and an overall age-standardized rate in East Africa of 3.8 cases per million population^24^; however, earlier studies suggested incidence in the range of 30 to 60 per million children.^26^ Given this range, we tested scenarios of 100 to 1000 cases diagnosed annually by liquid biopsy. Because the proportion of positive cases among the tested samples is unknown, we allowed for a range between 10% and 90%. We then estimated the per-sample cost for the resultant number of samples, using costs for the lowest-cost machine for that throughput (Miseq up to 18 samples per week, NextSeq using a P1 flowcell above 18/week) while holding all other costs constant.

## Results

Table 2 shows the total cost per case for histopathology and liquid biopsy diagnoses. The cost of the liquid biopsy was $525 more than the current histopathology because of costly equipment and reagents; however, the staff component was slightly lower because of high levels of automation in modern molecular biology equipment.

**Table 2:** Microcosting of histopathology and liquid biopsy diagnoses: cost per case (2024 values, Tanzanian Shillings and US dollars).

Cost component	Histopathology, TZS ($)	Liquid biopsy, TZS ($)
Tissue biopsy	166 263 (72.29)	-
Staff	76 468 (33.25)	60 160 (26.16)
Equipment	18 153 (7.89)	307 836 (133.84)
Consumables	121 426 (52.79)	993 131 (431.80)
Overhead	43 209 (18.79)	272 226 (118.36)
Total	425 520 (185.01)	1 633 353 (710.15)

TZS indicates Tanzanian Shillings.

### Histopathology

**Figure 1:**
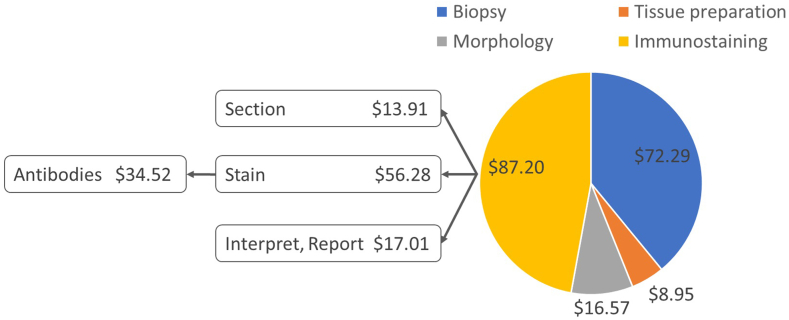
Costing of histopathology. Biopsy: collection of tumor tissue sample from patient; tissue preparation: grossing, fixing in formalin; morphology: sectioning, hematoxylin and eosin staining, interpretation and review; immunostaining: sectioning, preparing control sections, staining with antibodies, interpretation, and review. Morphology and immunohistochemistry costs both include tissue sectioning, staining, interpretation, and reporting—shown in detail for the immunohistochemistry.

Cost-per-case estimates for each stage are shown in Figure 1. The largest contributor is immunohistochemistry, driven predominantly by staining consumables ($43.49 of the $56.28 staining cost, accounting for 82% of all consumables), of which $34.52 are antibodies. Interpretation and reporting costs are largely time for review by 3 pathologists ($11.25).

In sensitivity analysis (see Appendix 5 in Supplemental Materials found at https://doi.org/10.1016/j.vhri.2025.101113) the main sensitivities are the number of immunostains per patient, cost of immunostaining reagents (antibodies and bulk reagents), and efficiency of utilization of the automated stainer for immunostaining. The total cost appears highly sensitive to the open biopsy cost because we incorporated the tariff cost rather than microcosting the biopsy process itself; therefore, it enters the estimate as a single, large number ($72.29). Further, MNH uses only 6.5% Trucut biopsies; hence, the limited sensitivity to the cost of Trucut biopsies in this setting.

### Liquid Biopsy

**Figure 2:**
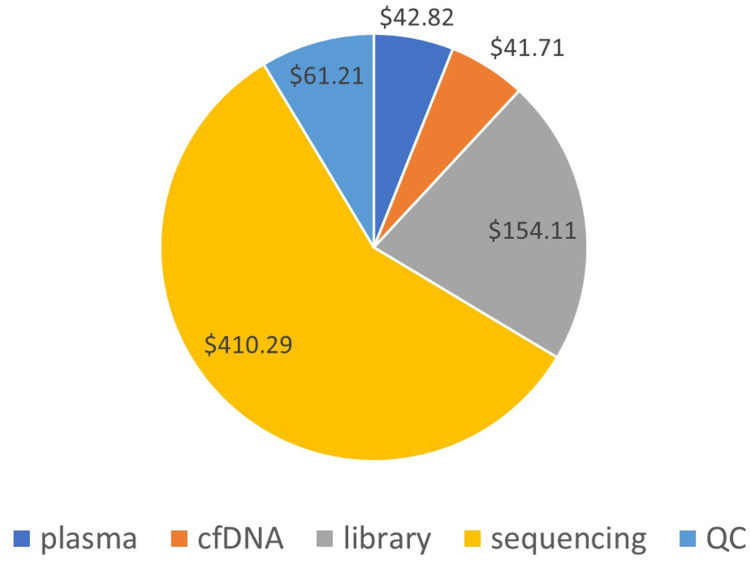
Costing of liquid biopsy. Plasma: blood collection and preparation of plasma from blood; cfDNA: isolation of cell-free DNA; library: amplification of DNA and hybridization capture of relevant sequences; sequencing: sequencing, analysis, and reporting; QC: checking of DNA concentration and the size of DNA fragments throughout the process. QC indicates quality control.

Cost estimates for each stage are shown in Figure 2. The largest contribution is the sequencing stage, which is because of the cost of the sequencing reagents ($175.48 per sample) and equipment ($49.91 per sample). Quality control steps include quantification of DNA concentration, and of the length of DNA fragments, both of which are required to allow technicians to adjust concentrations for subsequent preparation steps.

In sensitivity analysis (see Appendix 6 in Supplemental Materials found at https://doi.org/10.1016/j.vhri.2025.101113), the cost is sensitive to sample throughput, with the main effect being the number of samples per sequencing run, to share the cost of the sequencing reagents. A further decrease in total cost at 900 samples per year is mainly because of sharing laboratory equipment costs, notably the sequencing machine, across more samples. Other important sensitivities relate to the sourcing strategy for the DNA reagents, the size of shipments of reagents from Oxford, and the prices of those reagents. Laboratory efficiencies of maximizing the loading of the sequencing machine (multiplexing) and the bioanalyzer have an important effect on total cost. Decreased life of laboratory equipment results in an increase in overall cost. The apparently small effect of the dollar exchange rate is due to most reagents being purchased in sterling, which will already have absorbed the currency effects on reagents sold in US dollars.

### Scale-Up Scenarios

**Figure 3:**
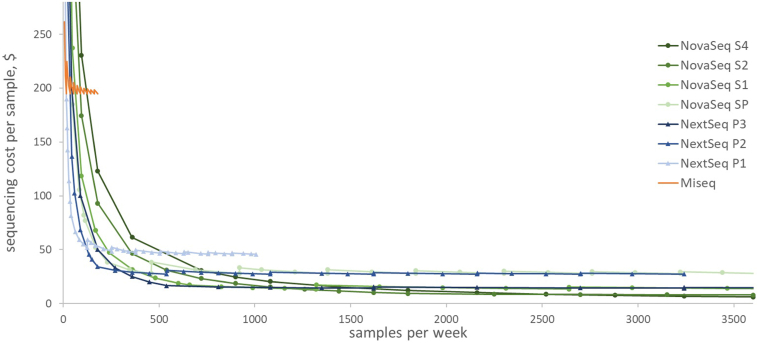
Cost per sample at increasing throughput. Cost in $ for the sequencing components (machine, reagents, and maintenance) for each machine at increasing throughput.

Cost estimates for the sequencing components of the liquid biopsy (sequencing machine, reagents, and maintenance) using alternative sequencing machines are shown in Figure 3. Below 18 samples per week, the MiSeq provides the lowest cost per sample, whereas larger machines are more expensive because of running under capacity. As the number of samples increases, the larger machines progressively become the lowest-cost option, and from 3000 samples per week (150 000 per year), the most expensive machine/flowcell combination is the lowest cost per sample. The steps in the curves are where an additional machine is required, resulting in a decrease in utilization and hence an increase in cost; the minimum cost per sample for each machine is the cost at maximum utilization.

### Cost Per Positive Case

**Figure 4:**
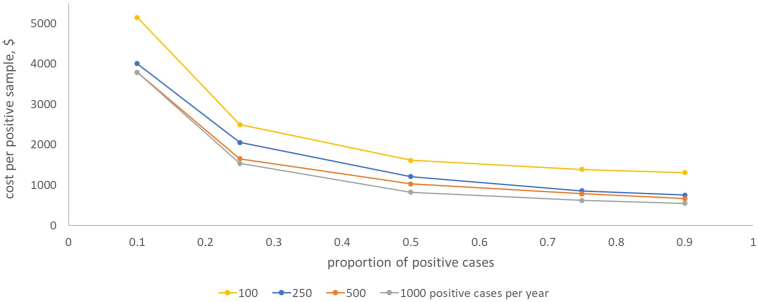
Cost per positive case detected. Cost ($) per case detected at varying levels of number of cases detected per year. Cost estimates are taken from the scale-up scenarios and use the cost estimate for the lowest-cost machine for the relevant number of samples (MiSeq up to 18 samples per week, NextSeq using a P1 flowcell thereafter).

The cost per positive case, at varying incidence and positivity rates, is shown in Figure 4. Although the number of cases, hence number of samples tested, affects cost, the much larger effect is the proportion of positive cases among the samples sent for analysis.

## Discussion

To our knowledge, this is the first microcosting of DNA sequencing performed in a laboratory in SSA. The per-patient cost of diagnosis of BL using panel sequencing is estimated at 1 633 353 TZS ($710.15), based on annual throughput of 300 samples. This is higher than diagnosis by histopathology (425 520 TZS, $185.01) at the current throughput of 40 daily samples. More than half the cost of the sequencing-based diagnostic is the sequencing part itself, which is because of the cost of the sequencing machine, its maintenance, and reagents. The cost is sensitive to sample throughput, reagent costs, and laboratory efficiencies. Costs are lower at a larger scale, using higher-capacity sequencing machines, provided that they are run at capacity. However, the cost per positive diagnosis will be highly dependent on the proportion of positive cases in the population of samples analyzed.

Compared with the previously published estimates of histopathology costs in this setting, our estimate was higher than the value found for Tanzania by Githang’a et al^27^ in their economic evaluation of childhood cancer treatment. They estimate average per-patient cost based on the number of tests performed and the hospital’s charge per test and report a 2016 value of $118 ($143 at 2024 values), including both pathology and laboratory tests. It may be that in 2016, the site under investigation was using predominantly morphology staining with little immunohistochemistry. Additionally, the site may not have had multiple pathologists available for second and third opinions nor scanning equipment to share slides with a network of pathologists in other hospitals, as applied in AI-REAL. Estimates for pathology costs in other sub-Saharan countries also date from 2015 to 2017 and range from $40 to $1251.^27^, ^28^, ^29^

General laboratory reagents and consumables used in these diagnostics are sourced locally, as are the specialist reagents for histopathology (antibodies). In contrast, the local supply chain for the specialist DNA reagents is limited. Hence, the study incurred additional costs of shipping these reagents from the United Kingdom. Building an independent local supply chain, with realistic costs that still allow the importing agent to cover their costs, remains a challenge for the sustainability of sequencing-based diagnostics in SSA. Increased throughput is expected to improve the viability of local supply, by enabling a local agent to import in larger quantities and reduce the contribution of dry-ice shipping costs.

Costs of both diagnostic methods are sensitive to the laboratory equipment lifespan. Maximizing the life of equipment requires effective machine maintenance, which proved challenging during the project, particularly for the relatively novel DNA sequencing equipment. Establishing local maintenance teams and affordable service contracts will be essential for manufacturers of sequencing technologies who wish to expand their businesses in this region.

The scale-up analysis indicates that future per-sample costs are expected to be dramatically lower, if increased throughput allows use of higher-capacity machines. However, higher throughput will require broadening beyond our current case study of BL, given the recent estimates of the annual incidence in Tanzania as 171 cases^25^—for example, to other lymphomas, early cancer detection, and infectious disease diagnosis and monitoring. Of note, some of the higher-capacity machines allow for several lanes in which different samples can be run. Therefore, it is not necessary to fill an entire run of the larger machines with 1 specific diagnostic, provided that there are users who can take the other lanes and therefore share the costs.

Our scale-up analysis assumes that all other cost components remain the same as in the base case. This may be conservative because there are likely to be economies of scale that we have not accounted for. For example, it is unlikely that the DNA and library preparation steps would be simply scaled up but would be redesigned to handle the large number of samples. Furthermore, at higher throughput, we expect that lower prices would be negotiated on reagents and maintenance contracts. However, the analysis assumes operation for 50 weeks per year; in reality, there would be downtime for maintenance and repairs, which would increase per-sample costs particularly where the machines are heavily used. We also assume that the sequencing machines are used most efficiently, running full as soon as sufficient samples are available; this may not fit readily into laboratory schedules.

The estimation of the cost per positive case depends on the number of positive cases and the positivity rate among the samples referred for sequencing. These, in turn, depend on the prevalence of BL, uptake of the liquid biopsy test by clinicians, and the choices of referring clinicians in which samples they refer. In the AI-REAL study, which recruited suspected lymphoma patients, around 25% were BL; therefore, a guideline to refer all suspected lymphoma patients for liquid biopsy might expect a positivity rate around this level. Both uptake and positivity rate are behavioral and therefore can be influenced by policy, education, and professional guidelines.

Liquid biopsy could be considered as an addition to, or replacement for, current histopathology. The current diagnostic algorithm for resource-limited settings^22^ is a staged protocol; a proportion of patients can be definitively diagnosed at each stage. If the liquid biopsy was inserted as an early stage in that protocol, this would avoid the need for further, relatively expensive, immunostains for confirmed BL patients. Therefore, the incremental cost of the liquid biopsy would be net of the savings in additional immunohistochemistry. Furthermore, the relatively low-cost morphology stain in stage 1 of the protocol could select suspected BL cases and therefore reduce the number needed to test per positive case; over 80% of BL cases show typical morphology at initial stain.^22^ Alternatively, the relative ease of taking a blood sample rather than a biopsy enables samples for liquid biopsy to be taken in local health centers, replacing histopathology for those patients diagnosed with BL. This would accelerate their diagnosis by up to a month,^8^ which would be expected to improve their prognosis and perhaps reduce the use of higher-cost second-line chemotherapy. The overall cost of the future testing pathway(s) will be affected by the sensitivity and specificity of the component tests and their position in the pathway, which will determine the total number of tests needed to arrive at a diagnosis.

Even if the liquid biopsy ultimately represents an increase in diagnostic cost, it could still be cost effective; this is predominantly a childhood cancer; therefore, any improvement in survival represents many life years gained. Realizing that survival benefit, however, requires that additionally diagnosed patients—whether through increased diagnostic sensitivity or improved access to diagnosis via local health centers—are able to access treatment. It remains to be seen whether current barriers to diagnosis persist as barriers to treatment (financial and geographical) despite the diagnosis. A full economic analysis is needed to evaluate overall cost-effectiveness.

There are several limitations of our cost analysis. First, the costs were estimated in the context of the AI-REAL study. For histopathology, this means that we costed the diagnostic process as implemented in the study, requiring the review of slides by 3 study pathologists. This requirement and the additional immunostains that are likely to have been ordered as a result may have increased the cost relative to standard practice in other sub-Saharan African sites. For the liquid biopsy, we costed the process of DNA preparation and sequencing in the research laboratory that developed this sequencing capability for during the study. This laboratory, therefore, was working at small scale, was still learning and improving, and was not designed as a service laboratory; these factors are likely to have biased cost estimates upward.

Second, specialist DNA reagents and consumables were supplied from the United Kingdom rather than from local suppliers. The costs are likely to change as the local supply chain is strengthened, supporting the increased uptake of these technologies. Third, the liquid biopsy costing is based on the Illumina platform; the findings may not generalize to alternative sequencing technologies. Fourth, for histopathology, we did not perform a microcosting of the tissue biopsy, instead using a tariff cost. This element has, therefore, not been costed in a consistent way with the rest of the work. Fifth, we accounted for overhead by assuming a constant rate of 20% of costs. Given the differing proportion of cost contributed by consumables in the 2 scenarios, this assumption may not hold. Sixth, we included donated equipment at the market cost. This inclusion will have increased our cost estimates relative to those accounted for in the laboratories. Finally, we have not estimated the costs of implementation for liquid biopsy. We have assumed that suitable laboratory space and trained staff will be available as they currently are for histopathology; there are likely to be costs incurred to reach this state.

## Conclusions

The cost of diagnosing BL using liquid biopsy and DNA sequencing is higher than current histopathology-based diagnosis. Both approaches are sensitive to throughput, costs of specialist reagents, and laboratory optimization. Costs are expected to fall considerably with increased scale, which is likely to require the expansion of the technology to a broader range of applications. This would be facilitated by development of local supply chain and maintenance capability. Despite the currently higher cost of liquid biopsy, it could still be cost effective because any improvement in survival in this childhood cancer represents many additional life years.

## Article and Author Information

**Author Contributions:***Concept and design:* Morrell, Schuh, Ruhago, Wordsworth

*Acquisition of data:* Morrell, Tungu, Achola, Legason, Magorosa, Mapendo, Mnango, Mremi, Christopher, Josephat, Burns, Dreau, Leonte, Chirande, Mahawi, Mkwizu, Mwamtemi, Sandi, Ruhago

*Analysis and interpretation of data:* Morrell, Tungu, Legason, Burns, Dreau, Leonte, El Mouden, Ruhago, Wordsworth

*Drafting of the manuscript:* Morrell, Tungu

*Critical revision of the paper for important intellectual content*: all authors

*Provision of study materials or patients:* Chirande, Mahawi, Mkwizu, Mwamtemi, Sandi

*Obtainingfunding:* Schuh, Ruhago, Wordsworth

*Administrative, technical, or logisticsupport:* Leonte, El Mouden

*Supervision:* El Mouden, Schuh, Ruhago, Wordsworth

**Funding/Support:** This research was funded by the National Institute for Health and Care Research (NIHR) (NIHR-RIGHT award 200133) using UK international development funding from the UK government to support global health research.

**Role of theFunder/Sponsor:** The funder had no role in the design and conduct of the study; collection, management, analysis and interpretation of data; preparation, review or approval of the manuscript; and decision to submit the manuscript for publication.

## Author Disclosures

Author disclosure forms can be accessed below in the Supplemental Material section.

The views expressed in this publication are those of the authors and not necessarily those of the NIHR or the UK government.

## References

[1] Crombie J., LaCasce A. (2021). The treatment of Burkitt lymphoma in adults. Blood.

[2] Gopal S., Gross T.G. (2018). How I treat Burkitt lymphoma in children, adolescents, and young adults in sub-Saharan Africa. Blood.

[3] Chamba C., Mbulaiteye S.M., Balandya E., Schuh A. (2023). Clinical application of circulating cell-free lymphoma DNA for fast and precise diagnosis of Burkitt lymphoma: precision medicine for sub-Saharan Africa. Camb Prisms Precis Med.

[4] Chantada G., Lam C.G., Howard S.C. (2019). Optimizing outcomes for children with non-Hodgkin lymphoma in low- and middle-income countries by early correct diagnosis, reducing toxic death and preventing abandonment. Br J Haematol.

[5] Minard-Colin V., Aupérin A., Pillon M. (2020). Rituximab for high-risk, mature B-cell non-Hodgkin’s lymphoma in children. N Engl J Med.

[6] Ozuah N.W., Lubega J., Allen C.E., El-Mallawany N.K. (2020). Five decades of low intensity and low survival: adapting intensified regimens to cure pediatric Burkitt lymphoma in Africa. Blood Adv.

[7] Joko-Fru W.Y., Parkin D.M., Borok M. (2018). Survival from childhood cancers in eastern Africa: a population-based registry study. Int J Cancer.

[8] Mawalla W.F., Morrell L., Chirande L.F. (2023). Treatment delays in children and young adults with lymphoma: report from an East Africa lymphoma cohort study. Blood Adv.

[9] Montgomery N.D., Liomba N.G., Kampani C. (2016). Accurate real-time diagnosis of lymphoproliferative disorders in Malawi through clinicopathologic teleconferences: a model for pathology services in sub-Saharan Africa. Am J Clin Pathol.

[10] Chamba C., Mawalla W. (2023). The future of lymphoma diagnosis, prognosis, and treatment monitoring in countries with limited access to pathology services. Semin Hematol.

[11] Legason I.D., Ogwang M.D., Chamba C. (2022). A protocol to clinically evaluate liquid biopsies as a tool to speed up diagnosis of children and young adults with aggressive infection-related lymphoma in East Africa (“AI-REAL”). BMC Cancer.

[12] Drummond M., Sculpher M., Claxton K. (2015).

[13] Xu X., Lazar C.M., Ruger J.P. (2021). Micro-costing in health and medicine: a critical appraisal. Health Econ Rev.

[14] Hamblin A., Wordsworth S., Fermont J.M. (2017). Clinical applicability and cost of a 46-gene panel for genomic analysis of solid tumours: retrospective validation and prospective audit in the UK National Health Service. PLoS Med.

[15] Schwarze K., Buchanan J., Fermont J.M. (2020). The complete costs of genome sequencing: a microcosting study in cancer and rare diseases from a single center in the United Kingdom. Genet Med.

[16] Sabatini L.M., Mathews C., Ptak D. (2016). Genomic sequencing procedure microcosting analysis and health economic cost-impact analysis: a report of the association for molecular pathology. J Mol Diagn JMD.

[17] Husereau D., Drummond M., Augustovski F. (2022). Consolidated Health Economic Evaluation Reporting Standards 2022 (CHEERS 2022) statement: updated reporting guidance for health economic evaluations. BMJ.

[18] Haacker M., Hallett T.B., Atun R. (2020). On discount rates for economic evaluations in global health. Health Policy Plan.

[19] Bank of Tanzania (Published 2023). Tanzania exchange rates. Bank of Tanzania.

[20] World Bank (Published 2024). Inflation, GDP deflator. https://data.worldbank.org/indicator/NY.GDP.DEFL.KD.ZG.

[21] Santos Gonzalez F., Mordaunt D., Stark Z., Dalziel K., Christodoulou J., Goranitis I. (2023). Microcosting diagnostic genomic sequencing: a systematic review. Genet Med.

[22] Naresh K.N., Ibrahim H.A., Lazzi S. (2011). Diagnosis of Burkitt lymphoma using an algorithmic approach-applicable in both resource-poor and resource-rich countries. Br J Haematol.

[23] Alaggio R., Amador C., Anagnostopoulos I. (2022). The 5th edition of the World Health Organization classification of haematolymphoid tumours: lymphoid neoplasms. Leukemia.

[24] Hämmerl L., Colombet M., Rochford R., Ogwang D.M., Parkin D.M. (2019). The burden of Burkitt lymphoma in Africa. Infect Agents Cancer.

[25] Mbulaiteye S.M., Devesa S.S. (2022). Burkitt lymphoma incidence in five continents. Haemato.

[26] Magrath I. (2012). Epidemiology: clues to the pathogenesis of Burkitt lymphoma. Br J Haematol.

[27] Githang’a J., Brown B., Chitsike I. (2021). The cost-effectiveness of treating childhood cancer in 4 centers across sub-Saharan Africa. Cancer.

[28] Renner L., Shah S., Bhakta N., Denburg A., Horton S., Gupta S. (2018). Evidence from Ghana indicates that childhood cancer treatment in sub-Saharan Africa is very cost effective: a report from the childhood cancer 2030 network. J Glob Oncol.

[29] Denburg A.E., Laher N., Mutyaba I. (2019). The cost effectiveness of treating Burkitt lymphoma in Uganda. Cancer.

