# Supplementary material for: Diagnosing Burkitt Lymphoma in Sub-Saharan Africa by Sequencing of Circulating Tumor DNA: A Comparative Microcosting Study
Source: Value Health Reg Issues. 2025 Jul;48:None. doi: 10.1016/j.vhri.2025.101113 (PMC12245731; doi:10.1016/j.vhri.2025.101113)
Supplement: Supplemental Material [file mmc2.pdf]

Diagnosing Burkitt Lymphoma in Sub-Saharan Africa by Sequencing of Circulating Tumour DNA: a Comparative Microcosting Study

Value in Health Regional Issues

Supplementary Material

Appendix 1: CHEERS checklist

Appendix 2: Data collection schematic overview

Appendix 3: Staff salary bands

Appendix 4: Sample capacity and costs of sequencing machines

Appendix 5: Sensitivity analysis – histopathology costing

Appendix 6: Sensitivity analysis – liquid biopsy costing

APPENDIX 1

CHEERS 2022 Checklist

Topic	No.	Item	Location where item is reported
**Title**			
	1	Identify the study as an economic evaluation and specify the interventions being compared.	Identified as a microcosting in the title; intervention specified in the title
**Abstract**			
	2	Provide a structured summary that highlights context, key methods, results, and alternative analyses.	Abstract
**Introduction**			
Background and objectives	3	Give the context for the study, the study question, and its practical relevance for decision making in policy or practice.	Introduction paragraphs 2-4
**Methods**			
Health economic analysis plan	4	Indicate whether a health economic analysis plan was developed and where available.	Costing framework is noted in Methods paragraph 2 and is available for reviewers; completed framework will be made available on publication
Study population	5	Describe characteristics of the study population (such as age range, demographics, socioeconomic, or clinical characteristics).	Introduction paragraph 3, Methods paragraph 1
Setting and location	6	Provide relevant contextual information that may influence findings.	Methods paragraph 1
Comparators	7	Describe the interventions or strategies being compared and why chosen.	Methods: Histopathology paragraph 1; Methods: Liquid biopsy paragraph 1
Perspective	8	State the perspective(s) adopted by the study and why chosen.	Methods paragraph 2
Time horizon	9	State the time horizon for the study and why appropriate.	Scope of microcosting noted in Methods: Costing approach paragraph 2
Discount rate	10	Report the discount rate(s) and reason chosen.	Methods: Costing approach paragraph 4

Topic	No.	Item	Location where item is reported
**Selection of outcomes**	11	Describe what outcomes were used as the measure(s) of benefit(s) and harm(s).	Not applicable
**Measurement of outcomes**	12	Describe how outcomes used to capture benefit(s) and harm(s) were measured.	Not applicable
**Valuation of outcomes**	13	Describe the population and methods used to measure and value outcomes.	Not applicable
**Measurement and valuation of resources and costs**	14	Describe how costs were valued.	Methods: Costing approach paragraphs 2-7
**Currency, price date, and conversion**	15	Report the dates of the estimated resource quantities and unit costs, plus the currency and year of conversion.	Methods: Costing approach paragraph 5
**Rationale and description of model**	16	If modelling is used, describe in detail and why used. Report if the model is publicly available and where it can be accessed.	No modelling is used
**Analytics and assumptions**	17	Describe any methods for analysing or statistically transforming data, any extrapolation methods, and approaches for validating any model used.	No transformations or extrapolation
**Characterising heterogeneity**	18	Describe any methods used for estimating how the results of the study vary for subgroups.	Single site costing so no heterogeneity characterised
**Characterising distributional effects**	19	Describe how impacts are distributed across different individuals or adjustments made to reflect priority populations.	Not applicable
**Characterising uncertainty**	20	Describe methods to characterise any sources of uncertainty in the analysis.	Methods: Sensitivity analysis
**Approach to engagement with patients and others affected by the study**	21	Describe any approaches to engage patients or service recipients, the general public, communities, or stakeholders (such as clinicians or payers) in the design of the study.	Method paragraph 1 - protocol reference
**Results**			

Topic	No.	Item	Location where item is reported
**Study parameters**	22	Report all analytic inputs (such as values, ranges, references) including uncertainty or distributional assumptions.	Table 1
**Summary of main results**	23	Report the mean values for the main categories of costs and outcomes of interest and summarise them in the most appropriate overall measure.	Table 2, Figure 1, Figure 2
**Effect of uncertainty**	24	Describe how uncertainty about analytic judgments, inputs, or projections affect findings. Report the effect of choice of discount rate and time horizon, if applicable.	Results: Histopathology paragraph 2; Results: Liquid biopsy paragraph 2; Appendix 2; Appendix 3
**Effect of engagement with patients and others affected by the study**	25	Report on any difference patient/service recipient, general public, community, or stakeholder involvement made to the approach or findings of the study	Not applicable
**Discussion**			
**Study findings, limitations, generalisability, and current knowledge**	26	Report key findings, limitations, ethical or equity considerations not captured, and how these could affect patients, policy, or practice.	Discussion
**Other relevant information**			
**Source of funding**	27	Describe how the study was funded and any role of the funder in the identification, design, conduct, and reporting of the analysis	Funding declaration
**Conflicts of interest**	28	Report authors conflicts of interest according to journal or International Committee of Medical Journal Editors requirements.	AAMC forms submitted for all authors

From: Husereau D, Drummond M, Augustovski F, et al. Consolidated Health Economic Evaluation Reporting Standards 2022 (CHEERS 2022) Explanation and Elaboration: A Report of the ISPOR CHEERS II Good Practices Task Force. Value Health 2022;25. doi:10.1016/j.jval.2021.10.008

APPENDIX 2

Data collection: schematic overview

1. Histopathology

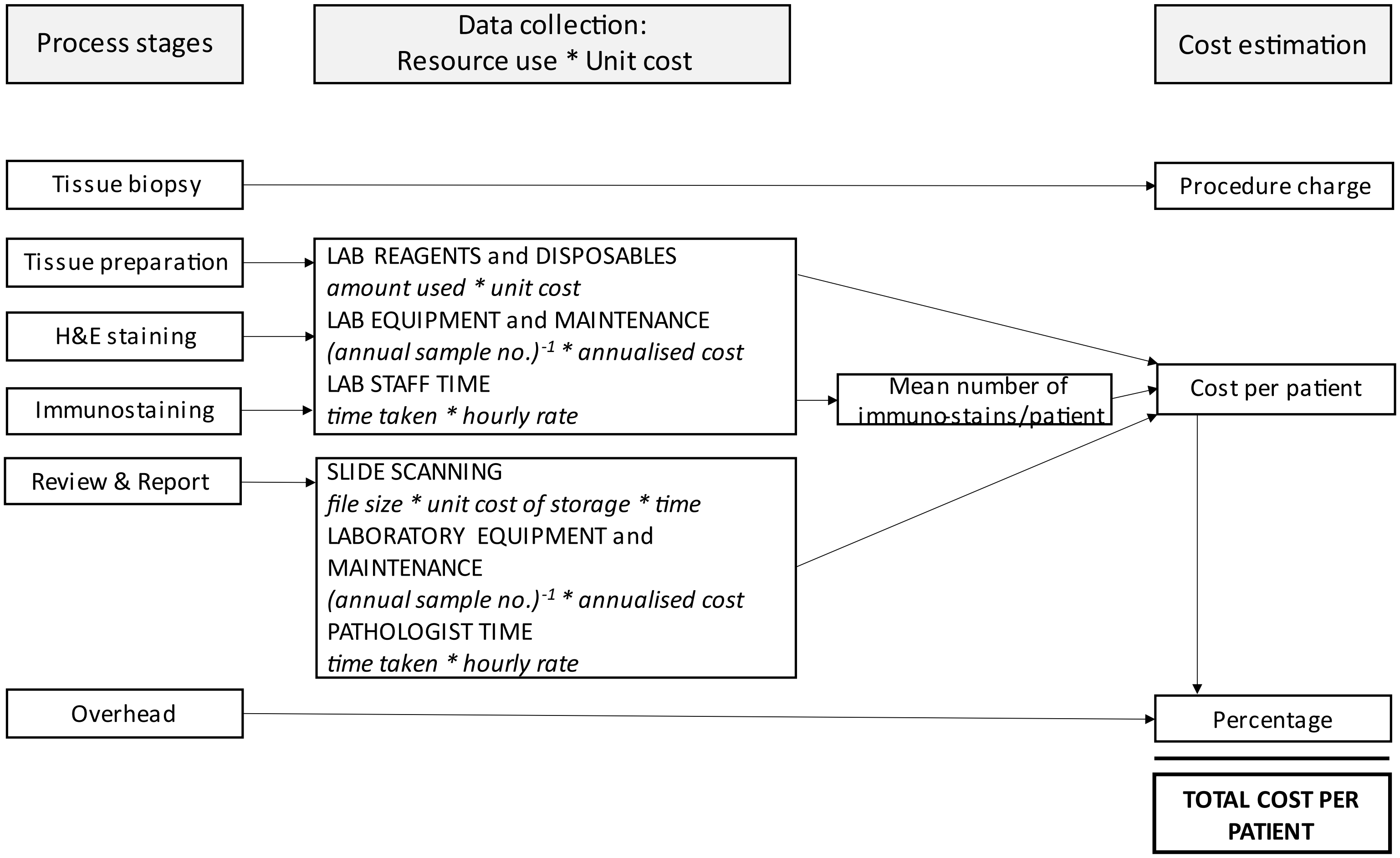

Resource use data are collected using resource use questionnaires for pathology laboratory staff. Questionnaire responses indicate whether each activity (and the resources used for it) is carried out on a day's batch of 40 samples together (for example, embedding in wax), or on an individual sample (for example, slicing with a microtome). Costs are calculated per H&E slide, and per immunostain, which is then multiplied by the average number of immunostains per patient from the AIREAL study.

The mean unit cost of antibodies for staining is derived from the specific antibodies used to diagnose patients in the AIREAL study.

Overheads are applied to the laboratory and review costs; the biopsy procedure charge is assumed to include an overhead element.

H&E stain: haematoxylin and eosin – a coloured stain that visualises the basic morphology of tissue's cells

Immunostain: visualising the amount and distribution of specific proteins in the tissue's cells using antibodies

2. **Liquid biopsy**

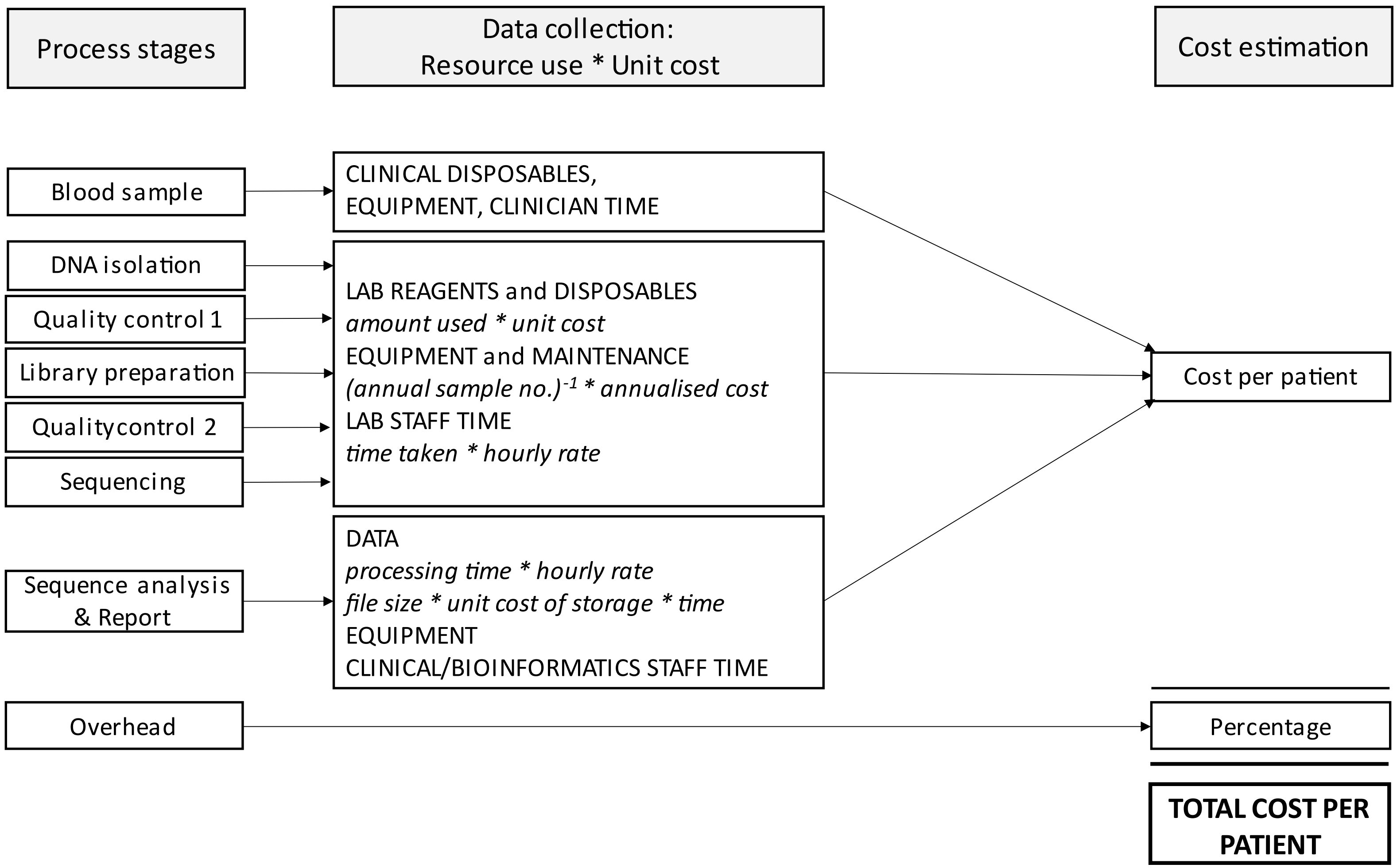

Resource use data are collected using resource use questionnaires for laboratory staff and clinicians. Questionnaire responses indicate whether each activity (and the resources used for it) is for an individual sample (for example, taking the blood) or for a group of samples being processed together (for example, isolating the DNA). Each sample represents one patient.

Overheads are applied across all process stages.

Library preparation: copying the DNA to generate sufficient material to work with, and selection of specific segments of the DNA for sequencing.
Quality control: quantifying the concentration and size of the DNA fragments in the sample, which are required to adjust concentrations for subsequent steps.

APPENDIX 3

Salary bands

1. Histopathology Reference year: 2021

		Monthly salary range (TSh)			Including social security/health insurance*			Cost/hr (TSh)#		
Job title	Band or Grade	Low	High	Mid-pt	Low	High	Mid-pt	Low	High	Mid-pt
Laboratory technician	PMGSS 5	977,000	1,067,000	1,022,000	1,104,010	1,205,710	1,154,860	6,419	7,010	6,714
Medical laboratory assistant	PMGSS 5	977,000	1,067,000	1,022,000	1,104,010	1,205,710	1,154,860	6,419	7,010	6,714
Biomedical scientist	PMGSS 7	1,780,000	1,954,000	1,867,000	2,011,400	2,208,020	2,109,710	11,694	12,837	12,266
Histotechnologist	PMGSS 8	2,041,000	2,224,000	2,132,500	2,306,330	2,513,120	2,409,725	13,409	14,611	14,010
Medical laboratory technologist	PMGSS 8	2,041,000	2,224,000	2,132,500	2,306,330	2,513,120	2,409,725	13,409	14,611	14,010
Medical laboratory scientist	PMGSS 10	2,630,000	2,960,000	2,795,000	2,971,900	3,344,800	3,158,350	17,278	19,447	18,363
Pathologist	PMGSS 17	4,780,000	4,910,000	4,845,000	5,401,400	5,548,300	5,474,850	31,403	32,258	31,831

2. Liquid biopsy Reference year: 2022

		Monthly salary range (TSh)			Including social security/health insurance *			Cost/hr (TSh) #		
Job title	Band or Grade	Low	High	Mid-pt	Low	High	Mid-pt	Low	High	Mid-pt
Junior clinician	TGHS D	1,100,000	1,215,000	1,157,500	1,243,000	1,372,950	1,307,975	7,227	7,982	7,605
Senior clinician (MNH)	TGS E - TGS F	2,900,000	3,800,000	3,350,000	3,277,000	4,294,000	3,785,500	19,052	24,965	22,009
Senior clinician (MUHAS)	PUTS 3.1 - PUTS 3.3	3,100,000	4,600,000	3,850,000	3,503,000	5,198,000	4,350,500	20,366	30,221	25,294
Lab technician (MUHAS)	MUHAS scale	1,000,000	2,000,000	1,500,000	1,130,000	2,260,000	1,695,000	6,570	13,140	9,855
Lab technician (MNH)	TGHS D	900,000	1,800,000	1,350,000	1,017,000	2,034,000	1,525,500	5,913	11,826	8,869
Technologist II	TGHS B	689,000	1,845,000	1,267,000	778,570	2,084,850	1,431,710	4,527	12,121	8,324
Bioinformatician	TGS D-TGS E	1,300,000	1,700,000	1,500,000	1,469,000	1,921,000	1,695,000	8,541	11,169	9,855
Medical admin/ lab attendant	TGS B-TGS C	420,000	510,000	465,000	474,600	576,300	525,450	2,759	3,351	3,055

		Annual salary range (GBP)			Including social security/health insurance **			Cost/hr (GBP) ##		
Job title	Band or Grade	Low	High	Mid-pt	Low	High	Mid-pt	Low	High	Mid-pt
Logistics manager, Oxford	Grade 5	£33,893	£44,509	£39,201	£33,893	£44,509	£39,201	£19.65	£25.80	£22.73

*	13%	TSh	Tanzanian Shillings
#	4.3 weeks per month, 40 hours per week	GBP	Great Britain pounds sterling
**	all employer costs included in the annual salary value		
##	46 weeks per year, 37.5 hours per week		

APPENDIX 4

Sample capacity and costs of sequencing machines

Machine and flowcell	Samples per run	Runs per week	Maximum samples per year #	Cost of machine	Cost of annual maintenance *	Cost of reagents
MiSeq	6	3	900	£75,000	£11,314	£675
NextSeq/P1	42	3	6,300	£250,000	£32,000	£1,100
NextSeq/P2	180	3	27,000	£250,000	£32,000	£3,000
NextSeq/P3	540	3	81,000	£250,000	£32,000	£5,000
NovaSeq/SP	230	2	23,000	£750,000	£75,000	£2,750
NovaSeq/S1	660	2	66,000	£750,000	£75,000	£4,500
NovaSeq/S2	1800	2	180,000	£750,000	£75,000	£8,250
NovaSeq/S4	3600	2	360,000	£750,000	£75,000	£12,000

Assuming 50 working weeks per year

* Maintenance cost for the MiSeq was the current annual cost for the MUHAS machine; as this cost was similar to that paid by Oxford Molecular Diagnostic Centre (OMDC), we used the OMDC cost for the NextSeq, and estimated the NovaSeq cost as a similar ratio (10%) of the purchase cost

APPENDIX 5

Sensitivity analysis – histopathology costing

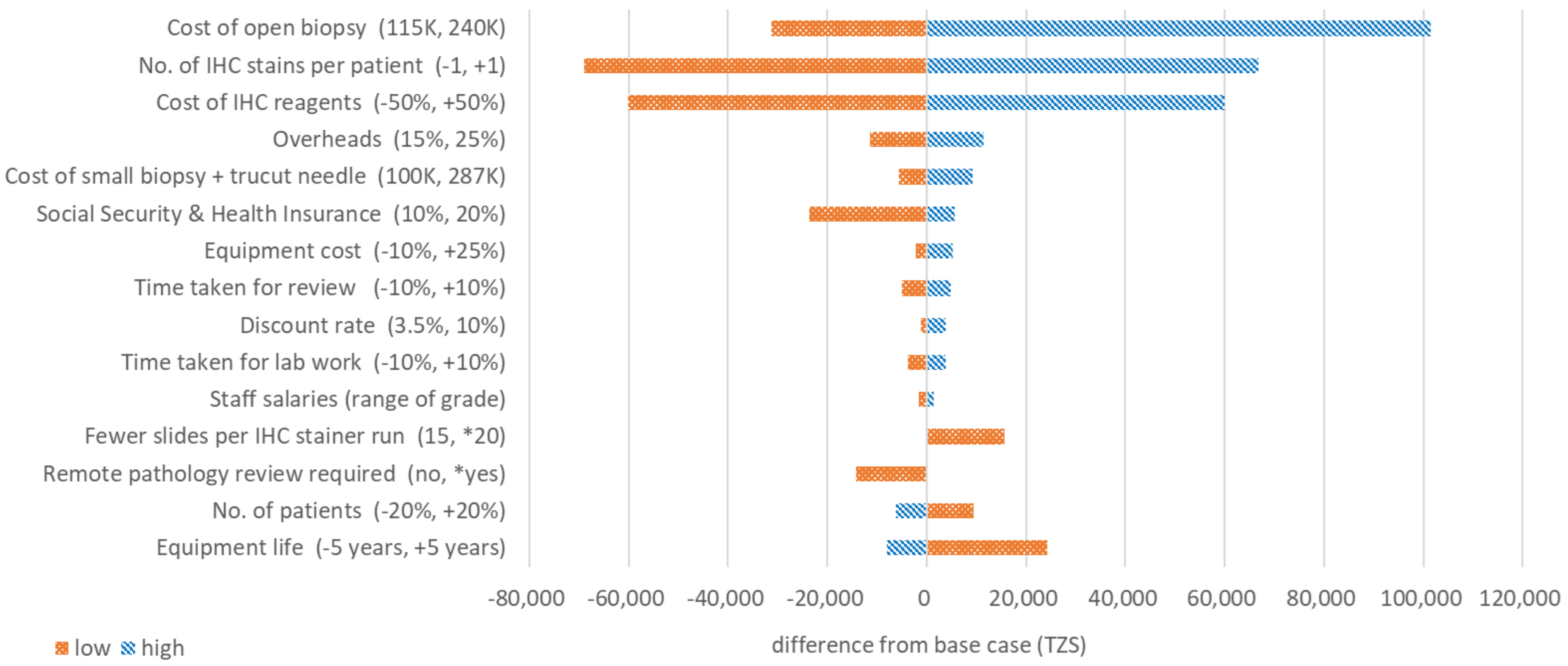

* base case

APPENDIX 6

Sensitivity analysis - liquid biopsy costing

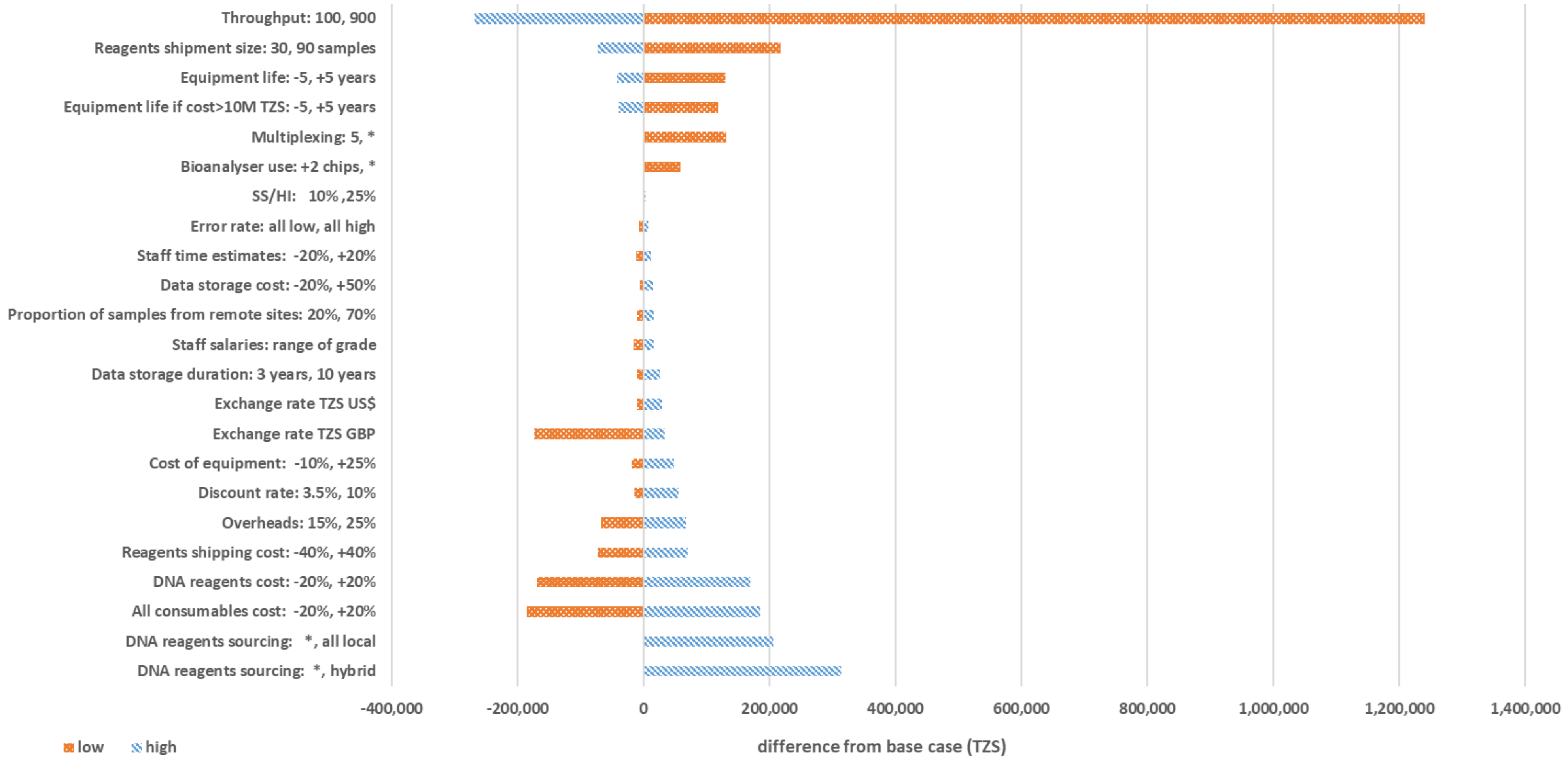

*   base case

Hybrid sourcing strategy – reagents for which we had a local price in October 2023 are assumed to be sourced locally, others shipped from Oxford

Local sourcing strategy – all reagents sourced locally with 50% price premium for DNA reagents relative to current, no shipping cost
